# Safety and tolerability of the olaparib tablet formulation in Japanese patients with advanced solid tumours

**DOI:** 10.1007/s00280-016-3106-7

**Published:** 2016-07-15

**Authors:** Kan Yonemori, Kenji Tamura, Makoto Kodaira, Koshi Fujikawa, Tamotsu Sagawa, Taito Esaki, Tsuyoshi Shirakawa, Fumihiko Hirai, Yuki Yokoi, Toshio Kawata, Ben Hatano, Yasuo Takahashi

**Affiliations:** 1 Department of Breast and Medical Oncology, National Cancer Center Hospital, 5-1-1 Tsukiji, Chuo-ku, Tokyo, 104-0045 Japan; 2 Department of Gastroenterology, National Hospital Organization Hokkaido Cancer Center, Hokkaido, Japan; 3 Department of Gastrointestinal and Medical Oncology, National Hospital Organization Kyushu Cancer Center, Fukuoka, Japan; 4 Department of Thoracic Oncology, National Hospital Organization Kyushu Cancer Center, Fukuoka, Japan; 5 AstraZeneca R and D, Osaka, Japan

**Keywords:** Olaparib, Poly(ADP-ribose) polymerase inhibitors, Clinical trial, Phase I, Safety, Solid tumours

## Abstract

**Purpose:**

This was the first Phase I study to assess the safety and tolerability of the tablet formulation of olaparib (Lynparza™), an oral poly(ADP-ribose) polymerase inhibitor, in Japanese patients with advanced solid tumours. The pharmacokinetic profile and antitumour activity of olaparib tablets were also assessed.

**Methods:**

In this open-label, multicentre study (D081BC00001; NCT01813474), a single dose of olaparib (200 or 300 mg, tablets) was administered on day 1, followed 48 h afterwards by multiple dosing (200 or 300 mg twice daily [bid]) for 28-day cycles. Doses were escalated in successive cohorts, with an expansion cohort enrolled at the highest dose that was confirmed to be tolerable during dose escalation.

**Results:**

Twenty-eight patients were enrolled and 23 were treated (*n* = 4, 7 and 12 at 200, 300 and 300 [expansion] mg bid, respectively). No patients experienced a dose-limiting toxicity, so the maximum tolerated dose was not defined. The most frequent adverse events were nausea (43.5 %), decreased appetite (30.4 %), anaemia (26.1 %) and constipation (26.1 %). No patient had dose reductions, two had dose interruptions, and two discontinued treatment because of adverse events. Absorption of olaparib was rapid following single and multiple dosing, and plasma concentrations declined biphasically after single dosing. No patients had a confirmed antitumour response.

**Conclusions:**

Olaparib tablet doses of 200 and 300 mg bid were considered tolerable in Japanese patients with advanced solid tumours. Consistent with the global olaparib programme, 300 mg bid was selected as the recommended tablet dose for future studies.

**Clinical trial registration number:**

NCT01813474.

## Introduction

Olaparib (Lynparza™) is an oral inhibitor of poly(ADP-ribose) polymerase (PARP) that, amongst other effects, blocks base-excision repair by trapping PARP at sites of DNA damage, leading to the collapse of DNA replication forks and the accumulation of DNA double-strand breaks [1]. PARP inhibition induces synthetic lethality in tumour cells that are deficient in pathways involved in the repair of DNA double-stranded breaks by homologous recombination repair, such as cells with *BRCA1/2* mutations [2, 3].

A Phase I monotherapy study established the maximum tolerated dose (MTD) of olaparib capsules as 400 mg twice daily (bid) [4]. Subsequent Phase II monotherapy studies have shown that olaparib is generally well tolerated at 400 mg bid, and antitumour activity has been consistently observed with this dose in patients with and without *BRCA1/2* mutations [5–8]. In patients with platinum-sensitive recurrent serous ovarian cancer, olaparib maintenance monotherapy significantly prolonged progression-free survival compared with placebo (hazard ratio [HR] 0.35, 95 % confidence interval [CI] 0.25–0.49, *P* < 0.001), and those with a *BRCA1/2* mutation were most likely to benefit from treatment (HR 0.18, 95 % CI 0.10–0.31, *P* < 0.0001) [9, 10]. The olaparib capsule formulation has received approvals at the 400 mg bid dose in the EU and US for the treatment of ovarian cancer.

Patients must take 16 × 50 mg capsules per day to reach the olaparib MTD, a ‘pill burden’ that may compromise patient convenience and compliance. This drove the development of a tablet formulation, designed to reduce the dose units required. However, as the tablet and capsule formulations are not bioequivalent, a simple formulation switch was not possible. Instead, a dose-finding study was conducted in Western patients with advanced solid tumours, which concluded that a 300 mg bid tablet dose (4 × 150 mg tablets per day) best matched the 400 mg bid capsule dose in terms of tolerability and efficacy [11]. This tablet dose is now being used in Phase III olaparib monotherapy studies.

However, there are currently no data for olaparib tablets in Japanese patients. Furthermore, in Western patients, exposure at the recommended 300 mg bid tablet dose exceeds that previously experienced by Japanese patients at the 400 mg bid capsule dose [11, 12]. Therefore, this Phase I study was conducted to assess the safety and tolerability of the olaparib tablet formulation in Japanese patients with advanced solid tumours.

## Patients and methods

### Patients

Eligible Japanese patients were aged ≥20 years, with advanced solid tumours that were refractory to standard therapies or had no available standard treatments; had Eastern Cooperative Oncology Group (ECOG) performance status 0–1; and had adequate organ and bone marrow function and a life expectancy of ≥16 weeks. *BRCA1/2* mutation status was not assessed or used as an eligibility criterion.

All patients provided informed consent. The study was conducted in accordance with Good Clinical Practice, the Declaration of Helsinki and the AstraZeneca Policy on Bioethics [13].

### Study design and treatment

**Fig. 1:**
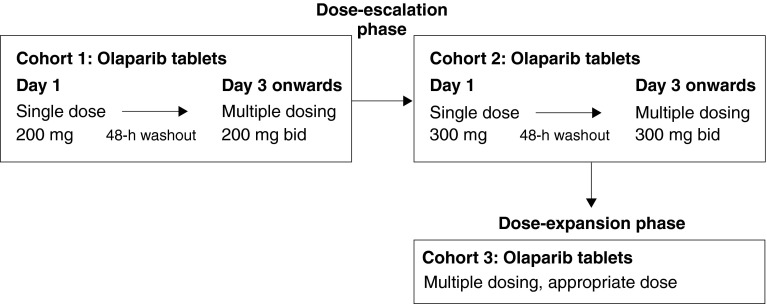
Phase I study design with dose-escalation and dose-expansion phases

This was a Phase I, open-label study (D081BC00001; NCT01813474) conducted across three Japanese centres. A dose-escalation scheme was used, with monitoring of safety and tolerability at each dose. The treatment regimen consisted of a single dose on day 1, followed by a 48-h washout and then continuous dosing for 28-day cycles (Fig. 1). A ‘rolling six’ cohort design was used, with 3–6 patients per cohort. The starting dose (cohort 1) was 200 mg bid and, if this was considered tolerable, the dose was escalated to 300 mg bid (cohort 2). A dose was considered non-tolerable if two out of six patients in the cohort experienced a dose-limiting toxicity (DLT). If the dose in cohort 2 was not tolerable, an intermediate dose between 200 and 300 mg bid could be investigated to identify the MTD. A dose-expansion phase was planned with a cohort of 12 additional patients for the highest dose that was confirmed to be tolerable during dose escalation. Doses of ≥400 mg bid were not investigated as, in Western patients, a tablet dose of ≥400 mg bid was not considered suitable for Phase III trials [11].

The primary objective was to investigate the safety and tolerability of escalating doses of the olaparib tablet formulation in Japanese patients with advanced solid tumours. Secondary objectives were to characterise the pharmacokinetic (PK) profile of olaparib tablets after single dosing and at steady state after bid dosing and to potentially establish the MTD. Antitumour activity was assessed as an exploratory objective.

### Assessments

Safety and tolerability were assessed by recording adverse events (AEs), physical examination, vital signs, electrocardiogram changes and laboratory findings. AEs were graded using the National Cancer Institute’s Common Terminology Criteria for Adverse Events (NCI CTCAE) v4.0 and were summarised from the first dose to within 30 days after discontinuation. A DLT was defined as any of the following events occurring during the first treatment cycle and determined by the investigator to be related to olaparib, irrespective of whether the event was resolved: absolute neutrophil count (ANC) < 0.5 × 10^9^/L for >5 days; ANC < 0.5 × 10^9^/L with neutropenic fever or sepsis; platelet count <25 × 10^9^/L; ≥2-week treatment interruption because of grade ≥2 anaemia and/or blood transfusion (in patients not requiring transfusion in the month before registration); non-haematological grade 3/4 toxicities (except fatigue, nausea, vomiting, diarrhoea, myalgia or arthralgia, unless prophylactic or therapeutic measures were administered for these); grade 2 cardiac or neurological toxicity; toxicity leading to treatment discontinuation in cycle 1; or any other toxicity judged by the investigator to be a DLT.

During the dose-escalation phase, blood samples for PK analysis were obtained before the single dose, at pre-defined intervals until 48 h after the single dose, before the morning dose on day 15 of the multiple-dosing period and at pre-defined intervals until 12 h after this dose. PK parameters derived following single dosing included maximal plasma concentration (*C*_max_), time to *C*_max_ (*t*_max_), terminal half-life (*t*_½*λz*_), area under the concentration–time curve (AUC), AUC from 0 to 12 h (AUC_0–12_), apparent plasma clearance (CL/F) and apparent volume of distribution (*V*_*z*_/F). Parameters derived following multiple dosing included *C*_max_ at steady state (*C*_ss, max_), time to *C*_*ss*, max_ (*t*_ss, max_), minimum plasma concentration at steady state (*C*_ss, min_) and AUC at steady state (AUC_ss_).

Tumour response was assessed by computed tomography or magnetic resonance imaging every 8 weeks until disease progression and graded using Response Evaluation Criteria in Solid Tumors (RECIST) v1.1.

### Statistical analyses

Safety data were summarised descriptively for the safety analysis set, which included all patients who received at least one dose of olaparib.

The PK analysis set included all patients from the dose-escalation phase who received at least one olaparib dose and had any evaluable post-dose PK data. PK parameters were derived using non-compartmental methods.

The efficacy analysis set included all patients who received at least one dose and had measurable baseline disease.

## Results

### Patient characteristics

Twenty-eight patients were enrolled (between 25 March and 31 October 2013) and 23 received treatment (*n* = 4, 7 and 12 in cohorts 1 [200 mg bid], 2 [300 mg bid] and 3 [300 mg bid expansion], respectively). Two patients were not eligible, two decided not to receive treatment and one withdrew from the study to undergo treatment for brain metastasis. Patient baseline demographics and characteristics (Table 1) were considered representative of the general study population. Disease classification included locally advanced and metastatic tumours and the median number of prior chemotherapy regimens was three (range 0–9). One patient did not have measurable baseline disease, so 22 patients were analysed for efficacy. All 11 patients from the dose-escalation phase (cohorts 1 and 2) were included in the PK analysis set.

**Table 1:** Patient demographics and baseline characteristics

	Cohort 1 200 mg bid (*n* = 4)	Cohort 2 300 mg bid (*n* = 7)	Cohort 3 300 mg bid (*n* = 12)	Total (*n* = 23)
Median age, years (range)	38.0 (37–55)	61.0 (44–64)	56.5 (34–77)	55.0 (34–77)
*Sex, n* (%)				
Male	0 (0.0)	3 (42.9)	5 (41.7)	8 (34.8)
Female	4 (100.0)	4 (57.1)	7 (58.3)	15 (65.2)
*ECOG performance status, n* (%)				
0	3 (75.0)	5 (71.4)	10 (83.3)	18 (78.3)
1	1 (25.0)	2 (28.6)	2 (16.7)	5 (21.7)
*Primary tumour location, n* (%)				
Breast	1 (25.0)	1 (14.3)	3 (25.0)	5 (21.7)
Ovarian	0 (0.0)	2 (28.6)	2 (16.7)	4 (17.4)
Lung	0 (0.0)	1 (14.3)	2 (16.7)	3 (13.0)
Other^a^	3 (75.0)	3 (42.9)	5 (41.7)	11 (47.8)
*Previous chemotherapy regimens, n* (%)				
0	1 (25.0)	0 (0.0)	1 (8.3)	2 (8.7)
1	1 (25.0)	2 (28.6)	1 (8.3)	4 (17.4)
2	0 (0.0)	1 (14.3)	1 (8.3)	2 (8.7)
3	0 (0.0)	1 (14.3)	6 (50.0)	7 (30.4)
>3	2 (50.0)	3 (42.9)	3 (25.0)	8 (34.8)

^a^Cohort 1: cervix (*n* = 2), uterus (*n* = 1); cohort 2: pancreas (*n* = 1), uterus (*n* = 1), retroperitoneum (*n* = 1); cohort 3: colon (*n* = 1), colorectal (*n* = 1), gastric antrum (*n* = 1), peritoneum (*n* = 1), primary location unknown (*n* = 1)

### Safety and tolerability

#### Exposure

The median (range) total treatment duration (including the 48-h washout and dose interruptions) was 72 (6–492), 30 (11–113) and 56 (39–289) days in cohorts 1, 2 and 3, respectively. The median (range) relative dose intensities were 97.1 % (88.9–100.0 %), 99.1 % (94.7–100.0 %) and 98.8 % (65.6–100.0 %) in cohorts 1, 2 and 3, respectively. After the data cut-off for the primary analysis (31 July 2014), two patients (cohort 1, *n* = 1; cohort 3, *n* = 1) continued to receive olaparib and 21 patients had discontinued treatment. Eighteen patients discontinued as a result of worsening of underlying disease, two discontinued because of AEs and one decided to withdraw from the study.

#### Dose-limiting toxicities

No patients in this study experienced a DLT. The 300 mg bid dose was therefore considered tolerable and chosen for the expansion cohort. As doses exceeding 300 mg bid were not assessed, the MTD in Japanese patients was not established.

#### Adverse events

Of the 23 patients who received olaparib, 21 experienced at least one AE (Table 2). The most frequent AEs were nausea (43.5 %), decreased appetite (30.4 %), anaemia (26.1 %) and constipation (26.1 %), all of which occurred more frequently at 300 than at 200 mg bid. No new types of AE were reported in the 300 mg bid expansion cohort. Eighteen patients experienced at least one AE that was considered causally related to olaparib by the investigator; the most common causally related AEs were nausea (34.8 %), decreased appetite (30.4 %) and anaemia (21.7 %). Decreased white blood cell or neutrophil count (both 13.0 %) were the most common grade ≥3 AEs. Three patients (42.9 %) from cohort 2 experienced grade ≥3 AEs that were considered causally related to olaparib (decreased white blood cell count, decreased neutrophil count and decreased lymphocyte count [*n* = 1]; anaemia [*n* = 1]; decreased white blood cell count and decreased neutrophil count [*n* = 1]).

**Table 2:** AEs of all grades occurring in >25 % of patients and AEs of grade ≥3 occurring in >10 % of patients, for each cohort

	Cohort 1 200 mg bid (*n* = 4)		Cohort 2 300 mg bid (*n* = 7)		Cohort 3 300 mg bid (*n* = 12)	
	All grades *n* (%)	Grade ≥3 *n* (%)	All grades *n* (%)	Grade ≥3 *n* (%)	All grades *n* (%)	Grade ≥3 *n* (%)
Any AE	4 (100.0)	1 (25.0)	7 (100.0)	3 (42.9)	10 (83.3)	1 (8.3)
Nausea	1 (25.0)	0 (0.0)	6 (85.7)	0 (0.0)	3 (25.0)	0 (0.0)
Anaemia	0 (0.0)	0 (0.0)	4 (57.1)	1 (14.3)	2 (16.7)	0 (0.0)
Decreased appetite	0 (0.0)	0 (0.0)	4 (57.1)	0 (0.0)	3 (25.0)	0 (0.0)
Decreased white blood cell count	0 (0.0)	0 (0.0)	3 (42.9)	2 (28.6)	2 (16.7)	1 (8.3)
Constipation	0 (0.0)	0 (0.0)	2 (28.6)	0 (0.0)	4 (33.3)	0 (0.0)
Diarrhoea	0 (0.0)	0 (0.0)	2 (28.6)	0 (0.0)	2 (16.7)	0 (0.0)
Dyspepsia	0 (0.0)	0 (0.0)	2 (28.6)	0 (0.0)	1 (8.3)	0 (0.0)
Proteinuria	0 (0.0)	0 (0.0)	2 (28.6)	0 (0.0)	0 (0.0)	0 (0.0)
Malaise	0 (0.0)	0 (0.0)	2 (28.6)	0 (0.0)	2 (16.7)	0 (0.0)
Decreased neutrophil count	0 (0.0)	0 (0.0)	2 (28.6)	2 (28.6)	2 (16.7)	1 (8.3)
Ileus	1 (25.0)	1 (25.0)	0 (0.0)	0 (0.0)	0 (0.0)	0 (0.0)
Pyelonephritis	1 (25.0)	1 (25.0)	0 (0.0)	0 (0.0)	0 (0.0)	0 (0.0)
Decreased lymphocyte count	0 (0.0)	0 (0.0)	1 (14.3)	1 (14.3)	0 (0.0)	0 (0.0)

Two patients had dose interruptions because of AEs (decreased platelet count in one patient [cohort 2]; pneumonia in one patient [cohort 3]) and two patients discontinued treatment because of AEs. One patient (cohort 2) discontinued because of decreased neutrophil and white blood cell counts, which were considered to be causally related to olaparib. One patient (cohort 1) discontinued because of a serious AE (SAE) of ileus, which was not considered to be related to olaparib and was the only SAE reported. No patients had dose reductions caused by AEs. There were no AE-related deaths; one patient (cohort 3) died as a result of worsening of their condition.

There were no clinically relevant treatment-related changes in vital signs, electrocardiogram parameters, physical observations or laboratory variables reported, although mild myelosuppressive effect and mild renal impairment were observed in one patient.

### Pharmacokinetics

**Fig. 2:**
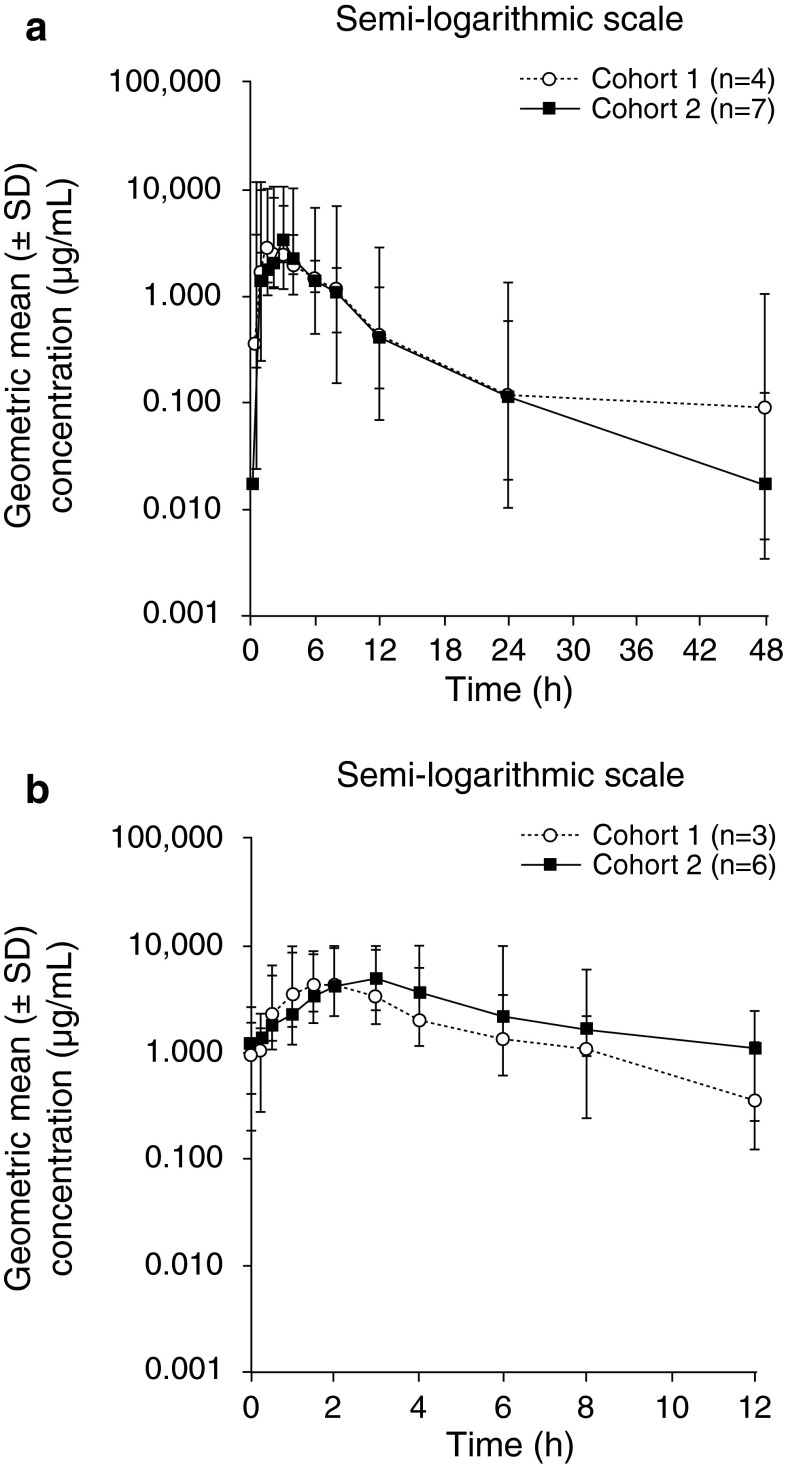
Plasma concentrations of olaparib following **a** single and **b** multiple dosing*. SD* standard deviation

Pharmacokinetic data were collected after single dosing for all 11 patients in the PK analysis set and after multiple dosing for nine patients (*n* = 3 in cohort 1; *n* = 6 in cohort 2). PK parameters are summarised in Table 3. Plasma-concentration–time profiles for single and multiple dosing are shown in Fig. 2; profiles were similar for the 200 and 300 mg bid doses. After single dosing, absorption of olaparib was rapid in both cohorts, with a median *t*_max_ of 2 h at 200 and 300 mg bid and a range of 1.0–3.0 h across cohorts. Plasma concentrations declined biphasically after the peak, with a mean *t*_½*λz*_ of 13.2 and 9.4 h at 200 and 300 mg bid, respectively, and a range of 6.5–18.6 h across cohorts. After multiple dosing, absorption was also rapid, with a median *t*_ss, max_ of 1.5 and 3.0 h at 200 and 300 mg bid, respectively, and a range of 1.0–3.9 h across cohorts. For both doses, median *t*_max_ did not differ significantly after multiple dosing compared with single dosing. Owing to a limited number of sampling points, *t*_½*λz*_ could not be adequately determined from the multiple-dosing profiles.

**Table 3:** Pharmacokinetic parameters for the tablet formulation of olaparib following single and multiple dosing for 15 days

PK parameters	Olaparib dose	
	Single dosing	
	Cohort 1 200 mg bid (*n* = 4)	Cohort 2 300 mg bid (*n* = 7)
*C*_max_, μg/mL	6.70 (95.69)	7.74 (34.76)
Median *t*_max_, h (range)	2.00 (1.00–3.00)	1.98 (1.00–3.00)
AUC_0–12_, μg h/mL	34.64 (159.7)	33.69 (34.52)
AUC, μg h/mL	61.97 (473.4)^a^	46.21 (64.64)
Mean *t*_½*λz*_, h (range)	13.24 (8.92–18.6)^a^	9.43 (6.45–14.7)
Mean CL/F, L/h (range)	6.38 (0.432–12.4)^a^	7.40 (2.29–13.3)
Mean *V*_*z*_/F, L (range)	93.96 (11.6–160)^a^	91.87 (47.8–151)

	Multiple dosing	
	Cohort 1 200 mg bid (*n* = 3)	Cohort 2 300 mg bid (*n* = 6)
*C*_ss, max_, μg/mL	7.67 (46.93)	8.43 (35.05)
*C*_ss, min_, μg/mL	0.61 (157.0)	1.29 (157.6)
Median *t*_ss, max_, h (range)	1.50 (1.00–3.00)	3.00 (1.50–3.93)
AUC_ss_, μg·h/mL	36.50 (71.94)	52.34 (68.17)

All values are given as geometric mean (CV %) unless otherwise stated

*CV* coefficient of variation

^a^It was not possible to calculate *t*_½*λz*_, AUC, CL/F or *V*_*z*_/F in one patient, therefore *n* = 3

Dose-normalised plasma exposures (*C*_max_ and AUC) were generally comparable between the 200 and 300 mg bid cohorts after single and multiple dosing. The exception was one patient (cohort 1) who showed significantly higher exposures after single dosing compared with the other patients; this patient withdrew from the study because of an AE (SAE of ileus) and was not included in the multiple-dosing analyses.

### Antitumour activity

Of the 22 patients analysed for efficacy, none had a confirmed complete or partial response. One patient in cohort 3 had an unconfirmed partial response at the first RECIST assessment; this patient had progressive disease (owing to the development of a new lesion) at the second RECIST evaluation. Overall, one (25.0 %), two (33.3 %) and four (33.3 %) patients in cohorts 1, 2 and 3, respectively, had stable disease for ≥8 weeks. Two (50.0 %), four (66.7 %) and eight (66.7 %) patients in cohorts 1, 2 and 3, respectively, had progressive disease. One patient (cohort 1) was not evaluable because of incomplete post-baseline assessments.

## Discussion

This was the first study to evaluate the safety and tolerability of the olaparib tablet formulation in Japanese patients. A Phase I dose-finding study has previously evaluated the olaparib capsule formulation in 12 Japanese patients with advanced solid tumours [12]. No DLTs were reported at an olaparib tablet dose of 200 or 300 mg bid, consistent with the previous observation of no DLTs at capsule doses up to and including 400 mg bid [12]. The 300 mg bid tablet dose was therefore considered tolerable and the MTD was not established, as higher doses were not investigated. AEs reported in this study were consistent with data for the tablet formulation in Western patients and with the known safety profile of the capsule formulation [4–7, 9, 11]. The most frequent AEs were nausea, decreased appetite, anaemia and constipation. No new AEs were observed in the 300 mg bid expansion cohort, and no new safety concerns were identified. AEs resulted in dose interruptions in two patients and treatment discontinuation in two patients; no patients required dose reductions because of AEs. The only SAE reported was by a patient with ileus of CTCAE grade 3, not considered causally related to olaparib, which resulted in treatment discontinuation. This patient showed significantly higher exposure to olaparib than other patients. Although the reason for this is unclear, it is possible that ileus may have affected olaparib absorption in this patient.

Absorption of olaparib was rapid following single and multiple dosing, with *C*_max_ reached by 1.00–3.93 h. The single-dosing profiles showed biphasic elimination, with a *t*_½*λz*_ of 6.45–18.6 h. The plasma-concentration–time profiles in this study were similar to those observed for the capsule formulation in Japanese patients and for the tablet formulation in Western patients [11, 12]. Following single and multiple dosing, exposure (*C*_max_, AUC) was similar to that for the tablet formulation in Western patients and exceeded reported exposure for the 400 mg bid capsule dose [11].

Objective responses were not seen in any patients. *BRCA* mutations were not evaluated in this study; therefore, future studies could investigate the efficacy of olaparib tablets in Japanese patients of known *BRCA* status.

In conclusion, the olaparib tablet formulation was tolerable in Japanese patients with advanced solid tumours and a dose of 300 mg bid is recommended for further evaluation. This is consistent with the tablet dose used in ongoing Phase III trials, such as GOLD (D081BC00004; NCT01924533), which will evaluate olaparib plus paclitaxel in Asian patients with advanced gastric cancer.
